# Clinical Instruction

**Published:** 1845-12

**Authors:** 


### CLINICAL INSTRUCTION.

The number of young physicians who resort to Paris to complete their medical studies, and the exaggerated accounts which are occasionally circulated of the great facilities there afforded for acquiring clinical instruction, has induced us to ask of Dr. Wilcocks, who has recently returned from Europe, an account of the institutions in that city most resorted to by students at the present time, and the manner in which the business is conducted. To this request he has kindly responded in the communication inserted on another page. It will be seen from that communication that the Dean of the Faculty, (Orfila) has issued an order which must very materially diminish the inducements heretofore presented by the French hospitals to students seeking instruction in practical medicine and surgery. It also appears that in other respects than that to which we have alluded, clinical instruction at Paris is conducted now just as it was twenty or thirty years ago, and is exactly the same as at the Pennsylvania Hospital in this city. The great difficulty there is in every one seeing the patient and profiting by the remarks of the prescriber, in the wards of a hospital, is a serious drawback on its advantages when the class in attendance is large, as at some of the Parisian hospitals, as well as at the Pennsylvania Hospital, where the class is still larger. To obviate this difficulty, in some degree, the Medical Colleges of Philadelphia have found it necessary to establish general Dispensaries in connection with their respective institutions, the patients of which are operated on and prescribed for in their large ampitheatres, in the presence of all the students. At these clinics, as they are called, the students witness the examination of the cases, hear the questions of the prescriber and answers of the patient; and after the patient retires or is removed from the ampitheatre, the Professor lectures on the case. That this is the best arrangement under the circumstances we have no doubt, and that the students find it conducive to their instruction is manifest from the fact that they are eagerly attended, day after day, by nearly a thousand of the pupils of the schools, besides physicians of the city.

The New York Medical Intelligencer contains some very appropriate observations on the subject of clinical instruction, extracted from an address by Dr. Corrigan, of Dublin, to which the editor appends the following remarks on the opportunities for hospital instruction afforded by the city of New York:

"These observations of Dr. Corrigan cannot be too strongly impressed on the minds of students of medicine. The advantages which the practice of a large hospital is capable of affording them, after two or three years' study of Anatomy, Physiology, and the other preliminary branches of a medical education, are incalculable. It is impossible to exaggerate, or overrate their value, and it is much to be regretted that there are not more facilities for obtaining them in connection with the various medical schools which have been established throughout the country. In New York we have two excellent Medical Colleges, each directed by professors of eminence and distinction, while we have only one hospital, and that not in any way connected with the teachers in either of the schools; for us to take any pains to show how much more interesting and efficacious the instructions of the Professors of Medicine and of Surgery would be, could they refer from time to time to cases under their care in the hospital, as practical illustrations of the doctrines they were desirous of enforcing, would be entirely superfluous. That this ought to be the case is self-evident. That it is not so requires no comment of ours, but we call upon the influential members of the profession who have its interests at heart, to use whatever power and authority they may possess to remedy a defect of so much importance, not only to the students of medicine, and to the character of the profession of which they are soon to become members, but also to the community at large, for the relief of whose ailments the profession itself has been called into existence. We are far from wishing to disparage in the slightest degree the talents and services of the present officers of the New York Hospital, but only regret that these have not been made available for the purposes of medical instruction. The primary purpose of the foundation of hospitals is, no doubt, for the relief of suffering humanity in the persons of the inmates; but we believe it is now universally conceded that such relief is not only perfectly compatible with a large amount of clinical instruction, but that its character is thereby elevated, and greater attention to the wants and comforts of the sick is thereby collaterally secured. It seems therefore in every way desirable that the Physicians and Surgeons of the Hospital should also be public instructors, whose services should be remunerated according to what would be, after all, the fairest test of their usefulness, the number of students that followed in their train, and attended their lectures. We think it important that they should be well paid for such services, for in the first place, students, generally, have no claim on them for gratuitous instruction; and in the second, it would be unreasonable to expect that men of eminence should devote so much of their time as is necessary fully to develope the resources of hospital practice, without adequate remuneration, and to the manifest neglect of their other more profitable engagements.

But even if all this were accomplished, and the Professors of Medicine and Surgery of one of the medical schools were each ex-officio one of the Physicians and Surgeons of the Hospital, there would still be wanting the stimulus of rivalry to keep up a spirit of emulation sufficiently active to ensure permanent excellence. The tendency of all monopolies is essentially to carelessness of the wants and interests of the public of whom they are independent; and we fear, therefore, that until there are more than one hospital, which surely the necessities of so populous a city might require, and the endowment of which the wealth of so great a commercial one might render a matter of little moment, the hospital instruction of New York will fail to rival that of London, or of Paris, or of Dublin.—*New York Medical Intelligencer*, Nov. 5, 1845.

